# Supplementary material for: Are carbon emissions trading and green financial instruments synergistic? -Comprehensive quantitative research based on content analysis
Source: PLoS One. 2024 Mar 7;19(3):e0298601. doi: 10.1371/journal.pone.0298601 (PMC10919601; doi:10.1371/journal.pone.0298601)
Supplement: S1 Data — (ZIP) [file pone.0298601.s001.zip › Data/地级市Z_F工作报告.zip!/║╙─╧/╨┼╤⌠╩╨/└┤╫╘╣1⁄2╓┌║┼í╛┬φ┐╦╩2╛▌═°í┐_2018.docx]

各位代表：

现在，我代表市人民政府，向大会报告工作，请予审议，并请各位政协委员和其他列席人士提出意见。

一、2017年以来工作回顾

2017年以来，全市上下以习近平新时代中国特色社会主义思想为指导，全面贯彻落实党中央、国务院和省委、省政府各项决策部署，开拓创新，克难奋进，经济社会发展呈现稳中有进、稳中向好态势。2017年生产总值实现2226.6亿元，增长6.7%；固定资产投资完成2415亿元，增长8.9%；社会消费品零售总额突破千亿元大关，增长10.6%；规模以上工业企业增加值增长4.5%。受非经济因素影响，以上四项指标未能实现预期，但我市经济发展的质量和效益不断提升。规模以上工业企业主营业务收入完成2545亿元，实现利润155.3亿元；一般公共预算收入突破100亿元、增长12.2%，其中税收收入67亿元、增长11.9%；万元生产总值能耗下降4.95%；服务业对经济增长的贡献率达58.3%；铁路、公路客货运周转量增速居全省前列；高新技术产业、五大主导产业对规模以上工业增长的贡献率分别达55.8%和60.4%；居民人均可支配收入增长10.3%；实际引进省外资金230亿元，增长8.6%，增速居全省第一。今年第一季度，全市经济发展实现开门红，生产总值达487.4亿元，增长8.4%，增速居全省第5位；1—5月，一般公共预算收入增长16.8%，社会消费品零售总额增长11.6%，规模以上工业企业增加值增长7.3%，固定资产投资增长7.9%。

（一）三大攻坚战扎实推进。防范化解风险。规范政府举债融资行为，积极化解企业风险，依法严厉打击非法集资，建立健全监管、预警和处理机制，守住了不发生区域性系统性风险的底线。聚力脱贫攻坚。贯彻精准方略，狠抓问题整改，完善责任体系，脱贫攻坚工作取得了决定性进展、实质性成果，年度综合考核位居全省第二方阵第一名，实现脱贫15.1万人，易地搬迁1.4万人，51个贫困村退出贫困序列，新县顺利通过贫困退出省级核查公示。建成“多彩田园”产业扶贫示范基地1610个，带动32万贫困人口增收。建立健全五道医疗保障线，在全省率先开展贫困家庭慢性病签约服务，贫困人口医疗费用综合报销比例在95%以上。交通扶贫、易地扶贫搬迁、旅游扶贫、电商扶贫成效显著，全省精准扶贫档案、交通扶贫、易地扶贫搬迁、旅游扶贫、电商扶贫和残疾人家庭无障碍改造等现场会在我市召开。狠抓环境治理。全力推进“三治标三治本”，去年PM10、PM2.5、二氧化硫、二氧化碳、一氧化碳五项因子浓度值均为全省最优，PM10、PM2.5同比分别下降6.2%和6.9%，优良天数同比增加29天，空气质量稳居全省首位；扎实推进水污染防治，建立市县乡村四级河长体系，去年全市河流断面水质整体达标，城市集中式饮用水水源水质达标率100%。今年前五个月，针对大气和水环境质量出现的新情况、新问题，采取针对性治理措施，初步遏制了下滑势头。启动土壤治理与修复，完成农用地土壤污染状况详查点位布设与核实工作。

（二）项目建设全面发力。扎实开展“项目建设年”活动，以项目补短板、增优势、强支撑。突出项目谋划。谋划“5818”扩大有效投资项目266个，总投资1291.2亿元、增长39%；省市重点项目187个，总投资1270.6亿元、增长近一倍。建立健全政府投资项目库，编制三年滚动投资计划，上报省级项目922个，总投资808.9亿元，项目梯次结构初步形成。建立专人专班，安排专项经费，强力推进重大项目前期工作。沿大别山高速明港至鸡公山段、淮滨至内乡高速豫皖省界至淮滨段纳入省前期工作，G107线绕市区段一级公路新建工程获省发改委核准，G312线绕市区段一级公路新建工程列入交通运输部“十三五”中期调整计划，京九高铁和宁西高铁信阳段争取工作得到省委、省政府大力支持，河南省大别山革命老区引淮供水灌溉工程规划顺利通过水利部审查，袁湾水库、张湾水库项目前期工作扎实推进。加快项目建设。“5818”扩大有效投资项目完成投资392.9亿元、省市重点项目完成投资405.8亿元，均超额完成年度投资计划。许昌至信阳高速信阳段、安阳至罗山高速上蔡至罗山段、新二十四大街沪陕高速下线至南京大道连接线改造工程开工建设，淮滨至信阳高速息县至邢集段顺利推进，G312线出山店水库淹没区段改线工程建成通车，出山店水库移民搬迁全部完成、大坝工程稳步实施，明港机场完成试飞和航线确定，淮河淮滨至息县航运工程淮滨段开工建设。破解瓶颈制约。坚持市级领导分包、重点项目建设例会和联审联批制度，完善考核评价机制。出台中心城区征地拆迁“两办法一方案”，30个重点项目累计拆迁房屋4866户，完成征地1.2万亩，没有发生因拆迁政策问题引发的影响社会稳定事件。推广PPP模式、利用政策性贷款、融资平台发债、基金引导、“三块地”改革等市场化手段，破解融资难题。64个项目进入财政部、省财政厅PPP项目意向库，9个PPP项目落地建设。加强用地保障，新增各类建设用地2.5万亩。大力实施“回归工程”，10.2万人回乡创办经济实体5.6万个，累计带动就业55万人；出台“信阳英才计划”，在北京、武汉、深圳等地招才引智。抢抓贫困地区“IPO绿色通道”政策机遇，引进胜高集团、赛思医疗等7家企业迁址我市。

（三）百城建设统筹提质。以百城建设提质工程为抓手，加速推进新型城镇化进程。注重规划引领。依据城市总规编制中心城区13个片区控规和19个专项规划，编制“两湖”区域、信阳空港经济区发展规划，完成中心城区总体城市设计及浉河两岸城市设计编制，分类推进村镇规划编制。统筹城乡发展。以老城改造为重点，统筹推进新区提升，新建、改造供水污水管网210.9公里、燃气管网129.4公里，建成绿地游园20多处，浉河公园等停车场建成投用，新改建城市公厕202座，是全省唯一荣获“全国厕所革命优秀城市奖”的地级市；新建大别山高中、羊山第三小学，稳步推进市中心医院羊山分院、市中医院高新区分院建设，市儿童医院病房楼已封顶，市第二人民医院主体楼竣工，全省首家民营三级医院河南圣德医院挂牌开诊，目前已设置床位2300张，和兴阳光城等商业服务设施建设顺利，新建信阳东站旅客换乘中心和2个公交场站，开工建设羊山森林植物园和羊山公园；实施火车站改扩建、新十八大街贯通、新七大道西延、新五大道下穿京广铁路、四一路立交桥拓宽改造、跨浉河三座大桥等工程，推动新老城区融合发展。注重产城融合，量身打造保税物流中心（B型）、新时代物流、铁路物流基地等一批轻资产、高附加值的龙头项目；围绕信阳高新区主导产业延链补链，引进天扬光电OLED显示屏、明阳智慧新能源装备产业园等一批重点产业项目；围绕羊山新区家居产业，打造泛家居产业集群，抢抓疏解北京非首都功能和沿海地区产业转移机遇，大力开展驻地招商，引进碧桂园现代筑美家居等一批行业龙头企业落户。积极推进百城建设提质试点县建设，淮滨县成功创建国家卫生县城，光山县成功创建河南省文明县城。

加快美丽乡村建设，累计创建人居环境整治达标村1319个、示范村672个，浉河区郝家冲村等76个村庄入选第一批国家级绿色村庄，平桥区郝堂村入选首批全国农村幸福社区，新县吴陈河镇、郭家河乡荣获2017年国家园林城镇称号。强化城市管理。城市综合执法改革职能划转走在全省前列，覆盖市县两级的数字化城市管理平台建成投用，全市域数字化城市管理模式基本形成。顺利通过国家卫生城市复审。

（四）转型发展步伐加快。以高端化、绿色化、智能化、融合化为目标，推进产业转型升级。发展特色农业。优质水稻占水稻种植面积90%以上，弱筋小麦占小麦种植面积30%以上，发展再生稻8.4万亩、稻渔综合种养27.5万亩。茶叶面积达210.8万亩，产值105亿元，“信阳毛尖”荣获生态原产地保护产品称号，位列中国茶叶区域公用品牌第二位，品牌价值63.5亿元。成功举办第26届信阳茶文化节。完成第三次全国农业普查，农村土地承包经营权确权登记颁证工作走在全国前列。培育省级以上农业产业化龙头企业60家，省级示范家庭农场17家，新增省级以上农民专业合作示范社21家。建成2个国家级、12个省级出口食品农产品质量安全示范区。商城县成功创建国家生态原产地产品保护示范区。发展新型工业。召开全市工业暨招商引资大会，以“五抓五提”为抓手，出台支持工业发展20条意见、招商引资奖励办法、产业集聚区建设奖惩办法等系列文件，建立重点企业“首席服务员”制度，推动形成大干工业、大抓招商的浓厚氛围。全市规上工业企业总数达1280家，新型建材产业规模突破600亿元，绿色食品产业规模突破800亿元，信阳高新区成为全省电子信息产业3个重点发展区域之一，全市百亿级产业集群达到8个，市商务中心区、浉河区特色商业区、平桥区产业集聚区晋升为省二星级。实施创新驱动发展战略，支持大众创业万众创新，新增2家省级创新龙头企业、15家高新技术企业，华英农业和毅辉智能制造、谷麦光电分别被认定为2017年河南省智能车间和智能工厂，国家级星创天地达到3家，国家级科技企业孵化器、众创空间实现零突破。积极开展“帮促”活动，深化放管服改革，去年共减免企业税费1.2亿元，市中小企业公共服务平台受理解决问题320个，被工信部授予2017年度国家中小企业公共服务示范平台。发展现代服务业。推进全域旅游，发展乡村旅游，建设环南湾湖茶旅融合精品线路，创建国家4A级景区3家、3A级景区4家，去年旅游综合收入226亿元，增长28.7%。新县被评为2017中国体育旅游目的地。加快发展电商物流业，我市被确定为全国农产品冷链流通标准化建设示范试点市，市跨境电商产业园正式运营，移联网信电子商务综合示范园落地息县，去年全市电子商务交易额130亿元、商品网上零售额93.7亿元，分别增长28.7%和46.5%。推动房地产业健康发展，完成投资431.6亿元，居全省第2位，拉动投资增长3.4个百分点。完善金融服务，农商行组建三年规划基本完成，中信银行、兴业银行落户我市，全市银行业金融机构达29家，存款、贷款余额分别居全省第4位和第6位；支持企业利用资本市场直接融资，新增融资69.7亿元，培育省定重点上市后备企业29家，新三板后备企业44家。

（五）民生福祉持续改善。全年累计民生支出346.9亿元，占财政支出的77.7%。办好民生实事。省重点民生实事全面完成。市十件民生实事社会反响良好：1、除中山中学因规划调整未开工外，其余6所学校新建、改扩建工程完成年度计划；2、中心城区4条断头路贯通工程全部开工；3、综合整治中心城区226条背街小巷，超额完成年度任务；4、中心城区公厕及垃圾中转站改造提升工程全部完工；5、浉河两岸排水口过渡性截污工程全部完成；6、标准化改造农贸市场25个，超额完成年度任务；7、互联网+明厨亮灶工程全部完成；8、全市农民自缴农业灌溉基本水费全部免除；9、提高全市城乡居民和城镇职工医保标准落实到位；10、清洁能源利用提速工程超额完成。发展社会事业。新建、改扩建幼儿园135所，实施中小学校舍维修项目304所，改造薄弱学校589所。高考成绩继续位居全省前列，空军招飞工作连续5年蝉联全国地市级首位，大别山艺术高等专科学校纳入河南省高校设置“十三五”规划，信阳航空职业学院挂牌成立，潢川县成功创建国家义务教育发展基本均衡县。公立医院药品零差价全面实行，分级诊疗扎实推进，两孩政策稳步实施，息县荣获省公立医院综合改革示范县。成功举办市第五届全民运动会。原创民族歌剧《八月桂花开》获得省第十一届精神文明建设“五个一工程”奖，罗山皮影戏赴法国巴黎专场演出。强化社会保障。城镇新增就业10.1万人，完成率居全省第一。机关事业单位养老保险制度改革、城乡居民医保整合和全民参保登记工作全面完成，城乡医保覆盖率达95%以上。残疾人“两项补贴”制度全面落实，率先在全省取消特困人员集中和分散供养标准差别，城乡居民最低生活保障标准分别提高到每人每年5160元和3210元。建成棚改安置房5530套，分配公租房4094套，完成农村危房改造12904户。持续深化平安信阳建设，加强食品药品安全监管，完善立体化社会治安防控体系，深入排查整治安全事故隐患，认真落实信访制度改革措施，人民群众安全感、满意度持续提升。

各位代表！本届政府履职一年多来，深入学习贯彻党的十九大精神，扎实推进“两学一做”学习教育常态化制度化；认真落实全面从严治党主体责任，严格执行中央八项规定精神，强化行政监察和审计监督，坚决纠正损害群众利益的不正之风；自觉接受人大、政协监督，办理人大代表建议235件、政协委员提案412件，及时办结率100%；建立健全“1211”依法行政推进机制，法治政府建设迈上新台阶；勇于解放思想，善于运用专班推进工作，政府投融资和资本运作取得突破，培养了一批懂业务的专业人才和本地项目推进团队，政府治理能力和治理水平不断提升。

切实加强精神文明建设，公民道德水平和城乡文明程度明显提升，我市保留全国文明城市提名资格，新县成功入选新一轮全国文明城市（县级）提名城市，7个村镇荣获第五届全国文明村镇，6所学校荣获首届全国文明校园。持续深化“双拥”工作，加强国防动员、国家安全、人民防空、民兵预备役、优抚安置等工作，实现河南省双拥模范城“七连冠”。驻信解放军和武警、消防部队在支持地方建设、维护稳定等方面作出了重要贡献，工会、共青团、妇联和科协、文联、社科联、红十字会等人民团体在经济社会发展中发挥了不可替代的作用，民族宗教、外事侨务、对台工作全面加强，物价、地质、气象、农机、档案、地方志、机关事务、防震减灾、无线电管理等工作取得了新的成绩。

各位代表！这些成绩的取得，得益于习近平新时代中国特色社会主义思想的科学指引，得益于党中央、国务院和省委、省政府的坚强领导，得益于市委统揽全局、科学决策，得益于市人大、市政协有力监督和大力支持，得益于全市人民团结拼搏、共同努力。奋斗历经艰辛，成绩来之不易。在脱贫攻坚的关键时期，广大基层干部、驻村第一书记忠诚履职，勤奋工作，增进了干部群众的血肉感情；在环境治理的攻坚阶段，全市各级攻坚战线、各位企业家、各个施工单位顾全大局，共克时艰，体现了令人敬佩的责任担当；在抗雪救灾的紧要关头，各级机关单位、广大党员干部紧急动员，抗灾减灾，保障了人民群众生命财产安全和正常生产生活。在此，我代表市人民政府，向全市广大工人、农民、知识分子，向解放军指战员、武警官兵、政法干警，向中直、省直机关和金融机构驻信单位，向各民主党派，向全市新闻工作者，向在我市创业的境内外投资者、建设者及社会各界人士，致以崇高的敬意和衷心的感谢！

在肯定成绩的同时，我们也清醒地认识到，我市发展不平衡不充分的问题还比较突出：一是经济总量小，产业结构不优，骨干企业少，产业集聚区等平台作用发挥不够。二是贫困人口基数大，全市还有26.6万贫困人口，脱贫攻坚任务依然繁重。三是公共服务均等化水平不高，城乡发展不平衡，城镇化水平低，民生保障能力有待加强。四是少数部门和政府工作人员担当精神不够，懒政怠政、执行不力和“四风”问题仍然存在，政府效能有待进一步提高。对此，我们将采取有力措施，认真加以解决。

二、2018年重点工作

2018年是贯彻落实党的十九大精神的开局之年，是改革开放40周年，是实施“十三五”规划承上启下的关键一年。党的十九大作出了中国特色社会主义进入新时代的重大判断，对推动经济由高速增长向高质量发展转变作出了全面部署。我们将认真学习贯彻习近平总书记系列重要讲话精神，落实好党中央国务院、省委省政府和市委各项决策部署，切实做好各项工作。

今年政府工作的总体要求是：全面贯彻落实党的十九大精神，以习近平新时代中国特色社会主义思想为指导，持续落实习近平总书记调研指导河南工作时的重要讲话精神，坚持党的领导，坚持稳中求进工作总基调，坚持新发展理念，紧扣社会主要矛盾变化，坚持高质量发展，统筹推进“五位一体”总体布局，协调推进“四个全面”战略布局，以供给侧结构性改革为主线，统筹推进稳增长、促改革、调结构、惠民生、防风险各项工作，加快建设现代化经济体系，着力打好“三大攻坚战”，着力打好“四张牌”，着力建设淮河生态经济带核心城市，着力构建“一市一区三枢纽”，着力建设“五个信阳”，深化“项目建设年”活动，开展“责任落实年”活动，推动信阳在全面建成小康社会、全面建设社会主义现代化新征程中实现跨越发展。

综合考虑，建议今年全市经济社会发展主要预期目标为：生产总值增长8.5%左右；规模以上工业增加值增长8.5%以上；全社会固定资产投资增长9.0%以上；社会消费品零售总额增长11%左右；进出口总值保持平稳增长；一般公共预算收入增长8.5%以上；居民人均可支配收入与经济增长同步；新增城镇就业9万人以上；城镇登记失业率4.5%以内；常住人口城镇化率提高1.5个百分点以上；居民消费价格涨幅3%左右；单位生产总值能耗降低6%以上，节能减排、环境保护完成省定目标。

围绕上述目标，重点做好以下九个方面的工作：

（一）坚持精准方略，坚决打好打赢脱贫攻坚战。坚持以脱贫攻坚统揽经济社会发展全局，将提高脱贫质量放在首位，围绕“两不愁、三保障”和“两率一度”集中总攻，以作风攻坚促进脱贫攻坚，确保固始县、光山县、潢川县、商城县、罗山县、息县脱贫摘帽和18.6万贫困人口脱贫、完成3997人易地扶贫搬迁。

聚焦贫困人口增收。因地制宜推进产业扶贫，建成“多彩田园”示范工程3000个以上，实现贫困村全覆盖；推广金融扶贫“卢氏模式”，探索政府、企业、银行、保险、贫困户“五位一体”金融扶贫新路径；推进绿色种养、乡村旅游等特色扶贫产业发展；支持龙头企业培育特色产业基地，吸纳贫困群众就业；加强贫困家庭劳动技能培训，力争让每个贫困家庭劳动力至少掌握一门技能、有一项稳定收入。加快补齐短板。将新增脱贫攻坚资金、项目向深度贫困村和“两非”地区倾斜，支持固始县打赢脱贫攻坚翻身仗。完善医疗扶贫，筑牢五道医疗保障线，全面推行轻度慢性病医疗救助，推进“一站式”即时结算，解除贫困群众看病之忧；加强教育扶贫，实现建档立卡贫困家庭学生全覆盖；加快农村危房改造及易地扶贫搬迁安置点建设，实现拟摘帽贫困县危房全部清零。着力推进村庄整治，全面完成贫困村水、电、路等基础设施及公共交通、广播电视、文化服务中心等基本公共服务建设。激发内生动力。坚持扶贫同扶志扶智相结合，引导贫困群众通过辛勤劳动实现脱贫。发挥示范引领作用，打造一批脱贫致富示范村、示范点，培育一批示范户。改进工作方式方法，将正面宣传与反面教育相结合、适当激励与监督约束相结合，营造勤劳致富、脱贫光荣的良好社会风尚。加强作风建设。狠抓脱贫攻坚作风建设年活动，压实行业部门帮扶责任，加大培训力度，打造懂扶贫、会帮扶、作风硬的扶贫干部队伍。集中力量解决扶贫领域形式主义、官僚主义等突出问题，坚决依纪依法惩治贪污挪用、截留私分、虚报冒领扶贫资金等行为。

（二）突出项目建设，着力增强发展支撑。深化“项目建设年”活动，加大招商引资力度，持续推进重大基础设施项目，重点推进产业类和民生类项目，以高质量项目促进高质量发展。

强化项目谋划储备。增强项目意识，紧跟政策趋向，提升谋划质量，确保谋划项目1000个、总投资6000亿元以上，全市项目储备库投资动态保持在1万亿元以上，其中工业项目2000亿元以上。做深做细项目前期，力争一批重大项目进入省项目库和中央预算内投资，全年争取上级资金30亿元以上。成立项目谋划领导小组，推进项目谋划常态化。启动濮阳至阳新高速公路商城至豫皖省界段、安阳至罗山高速罗山至豫鄂省界段、沿大别山高速鸡公山至商城段项目前期工作，争取淮滨至内乡高速豫皖省界至淮滨段年内开工。规划论证“两湖”大道并开展前期工作。制定市中心城区调水方案，把南湾湖、出山店水库、北湖和其它城市内河连为一体，推动市区水系建设。继续争取京九高铁信阳段“十三五”开工建设、宁西高铁信阳段项目启动前期工作。加快袁湾水库、张湾水库前期工作，力争完成河南省大别山革命老区引淮供水灌溉工程可研审批。分类推进项目建设。全年力争实施省市重点项目300个以上，年度完成投资700亿元。加强项目调控，建立政府投资类、产业发展类项目推进机制。突出政府投资类项目的引导作用，续建淮信高速息县至邢集段、淮河淮滨至息县航运工程淮滨段、G328固淮交界至淮滨县城段改建工程、淮河干流一般堤防加固工程信阳市段、“引鲇入固”饮水工程等项目；加快推进许昌至信阳高速公路信阳段、安阳至罗山高速上蔡至罗山段、河南五岳抽水蓄能电站等项目；完成黄缘闭壳龟省级自然保护区调整，开工建设沿大别山高速明港至鸡公山段、G107和G312线绕市区段一级公路新建工程。突出产业发展类项目的支撑作用，重点推动淮滨服装加工贸易基地、舜宇光学产业园等谋划实施，加快固始县南山25万千瓦风电一期、光山静脉产业园、谷麦光电科技产业园等项目建设，推进天煜光电OLED6代柔性屏等项目投产达产。全力抓好招商引资。强化产业招商，分产业制定招商规划，精准开展招商活动；强化园区招商，加强与国内外相关产业园区的对接，力促中电光谷信阳产业园落地，引进绿地集团参与高铁站片区开发；强化重点区域招商，精准对接京津冀地区企业，在长三角、珠三角设立常驻招商队伍，深入研究武汉、合肥等地产业关联度，积极融入“三区一群”发展战略。探索招商外包服务新模式，开展委托招商。大力实施“回归工程”，发展回归经济，开展信商大走访，充分发挥驻外招商办和商会作用，通过“回归工程”引进投资200亿元以上。持续利用贫困地区“IPO绿色通道”政策，积极引进拟上市优质企业。完善驻地招商和招商项目谋划机制，开展项目落地综合督导，形成全链条招商、全过程服务、全要素保障的招商引资工作体系。

（三）加快产业转型升级，调整优化经济增长结构。开展重点产业攻坚，明确时间表、路线图，确保规上工业企业主营业务收入突破2800亿元，第三产业增加值增长10%以上。

做大做强新型工业。持续实施“五抓五提”，坚定不移大干工业，推动工业向产业链和价值链高端延伸。加快传统支柱产业脱胎换骨式改造，实施食品、建材、纺织服装、装备制造4个传统支柱产业转型升级专项行动。食品产业方面，围绕米面肉油茶酒等，加强品牌建设；建材产业方面，聚焦新能源和装配式建筑领域，加快上天梯非金属矿产品研发推广，推动恒信汽车新材料、新联智能交通产业园等项目建设，延伸钢铁加工产业链，发展壮大摩根电梯、碧桂园现代筑美家居等家居产业；纺织服装业方面，抓好商业模式创新，打造全国最大的羽绒生产和羽绒制品基地；装备制造业方面，推动淮滨造船业军民两用技术双向交流转化，扩大开源环保公司环保设备市场占有率，培育装备制造零部件专业化公司。加快新兴产业创新赶超，围绕电子信息、高端装备制造、新能源、军民融合等产业，推进信阳高新区、平桥区、罗山县等电子信息产业园区联动发展，以天扬光电和舜宇光学为龙头，打造光电产业园和光学产业园，尽快形成液晶显示、光学镜片规模产能；加快建设明阳智慧新能源装备产业园，打造新能源装备生产基地；完成浩融集团新能源产业园项目一期工程，筹建超级电池电容原材料生产基地；推进毅辉智能制造、淮滨造船、694厂、信阳航空职业学院与军工央企对接合作，加快形成军民融合示范园区。推进绿色化、智能化和企业技术改造，实施“三大改造”项目308个，完成年度投资250亿元以上，建设绿色化示范工厂1家、智能工厂10个、智能车间10个，推动30家企业实施智能化改造。提升产业集聚水平。实行月考核、季观摩、年度奖惩，强力推进产业集聚区提质转型创新。启动全市产业集聚区控详规修编，统筹推进建成区、发展区、控制区功能布局调整。制定主导产业集群化发展举措，开展产业链协同创新、产业集群专题招商，每个产业集聚区至少招引1个亿元以上项目。加强厂房、设备、土地等低效闲置资产分类处置。建立“区域评价”“容缺办理”等机制，开展企业投资项目承诺制、政府投资项目联审制试点。规范产业集聚区管理机构和职能设置，下放市级政府经济管理权限。支持潢川县、潢川经开区融合创建国家级经济技术开发区。培育壮大骨干企业。支持非公经济发展，推动民营企业建立现代企业制度，引导企业集聚优势资源，进入资本市场。支持信钢、华英、羚锐、天扬光电、明阳智慧新能源5个现有企业打造百亿级龙头企业；扶持黄国粮业、春泉园林、胜高集团等12家企业上市筹备；助推枫叶园林、灵石科技、麦得隆食品、禹王水工等18家企业在新三板挂牌。支持中小企业走“专精特新”之路，打造一批专业化“小巨人”企业。做好“入规纳统”工作，新入库规模以上工业企业30家以上。完善重点企业、重点项目和重点产品定期调度机制，确保全年工业投资增长6%以上。

加快发展现代服务业。以旅游、康养、物流产业为重点，促进服务业实现速度质量双提升。着力发展现代旅游业。编制全域旅游发展规划，力争创建5A级景区1家，新增4A级景区2家。持续打造“三湖”区域旅游带，串联“三山两湖”旅游景区。推进茶旅融合，建成环南湾湖茶旅融合精品线路；持续实施“品信阳菜、喝毛尖茶”工程，推广“名菜、名吃、名厨、名店”。加强绿色生态旅游发展，实施南湾开元生态旅游、鸡公山建业天明文化旅游、浉河区观谷活力小镇、罗山县灵山特色小镇、光山大苏山森林公园、固始白鹭湖休闲旅游等项目；加快建设全国知名的红色旅游目的地，建好“重走长征路”等国家红色旅游精品线路；促进乡村旅游提质升级，培育5个乡村旅游示范乡镇（村），每个县区打造10个乡村旅游特色村和一批休闲农业产业园区，全年实现接待游客3900万人次、旅游综合收入290亿元以上。积极发展康养产业。以“两湖”区域为重点，加快培育以健康养生、旅游休闲、文化体育、科教培训等为主要内容的幸福产业，开展国家生命健康产业创新示范区前期工作；持续推进中心城区集中供热工程，完成和兴阳光城城市综合体一期建设，提升康养产业发展服务配套能力。大力发展现代物流业。开工建设林安物流智慧产业园，打造全市物流信息平台、货运平台、物流金融平台、仓储平台，建设豫南地区重要的电商物流分拨中心和最大的冷链物流基地；积极推进明港航空物流、淮滨水运物流集散中心、固始航运物流等工程，加快构建多式联运的物流体系。新建370个农村电商物流综合服务站点，实现省级电商进农村示范县全覆盖。

（四）推进百城建设提质，持续提升城市发展内涵。把百城建设提质工程与文明城市创建结合起来，统筹抓好规划、建设、管理、经营，不断提升城市品质。

完善城乡规划体系。坚持和完善规划委员会制度，突出“高精美特”规划标准，做好“以绿荫城、以水润城、以文化城、以业兴城”四篇文章，实现中心城区控规全覆盖。加快城市地下空间开发利用、绿地系统、停车设施、中小学及幼儿园等专项规划编制和报批。推进光山县、淮滨县、固始县专项规划编制，全面启动其他县的村镇体系规划修编工作。完成传统村落和历史文化名镇（村）保护规划编制。统筹城市建设。持续推进“政府主导、平台主建、部门监管、辖区拆迁”的工作机制，加强基础设施和公共服务配套设施建设。加快中心城区老城改造，做好新华路与四一路交叉口等拓宽渠化改造，提升改造建设东路、民权南路等一批老旧道路；推进南湾、湖东水厂联网循环供水，全面启动智能供水系统建设；加强内河两岸景观绿带、街头小游园和绿地建设。完善新区功能，加快建设大别山艺术高等专科学校、羊山第二小学、中心医院羊山分院、儿童医院、中医院高新区分院等一批教育医疗设施；持续推进“一校三馆”建设；完成羊山森林植物园、羊山公园主体工程，开工建设和园等城市公园。畅通中心城区新老城衔接，力争完成信阳火车站北广场区域改扩建、新十八大街贯通、新二十四大街改造、新七大道西延、新五大道下穿京广铁路、四一路桥涵拓宽改造工程；加快建设新十八大街南段跨浉河桥、浉河公园步行桥、浉河二号桥。提升县城和小城镇建设水平，加快中心城区“6+3”城市组团发展。深化文明城市创建。坚持领导带头、大员上阵，发挥基层网格作用，完成全国文明城市创建年度任务。认真落实市容和环境卫生管理条例，广泛开展“城市清洁”行动，深入开展交通秩序专项整治，营造干净整洁的城市环境。依法整治城市“三违”，坚决杜绝新“三违”现象发生。加快智慧城市建设，纵深推进城管执法体制改革，建立“协同联动”的指挥平台，提升城市精细化管理水平。以浉河新八景为载体典藏文化记忆，以百花园区域为窗口弘扬社会主义核心价值观，丰富城市人文内涵。加快推进诚信制度化建设，积极争创全国社会信用体系建设示范城市；大力开展志愿者服务，强化社会责任意识、规则意识、奉献意识，实现城市建设和市民素质双提升。

（五）实施乡村振兴战略，推进“三农”工作再上台阶。扎实推进“五个振兴”，完成乡村振兴总体规划和专项规划方案编制，逐步实现农业强、农村美、农民富。

提升农业发展质量。落实粮食安全行政首长责任制，新建高标准粮田60万亩，农业机械化水平提高到78%以上。持续调优种养结构，发展优质水稻650万亩、弱筋小麦160万亩、油茶80万亩、中药材4万亩、稻渔综合种养30万亩、水产养殖100万亩。持续调响特色品牌，叫响“信阳毛尖茶”区域公用品牌，扩大对外贸易，实现产值110亿元；加快农产品质量和食品安全标准体系及追溯体系建设，加强“三品一标”认证，推进国家生态原产地产品保护，进一步提升“信阳菜”“南湾鱼”“华英鸭”“固始鹅”等品牌知名度。持续调大经营规模，力争新发展示范家庭农场和农民专业合作社各30家、市级农业产业化重点龙头企业20家；引导农户采取出租、入股、转让、互换等方式加速土地流转。持续调长产业链条，推进农产品精深加工及农副产品综合利用，推进农业产业化集群发展和产业链延伸，建立健全农业支持保护体系，新增农业产业化集群7个。支持华英集团实施“1338”战略，打造种养加、产供销、内外贸一体化的农业产业化龙头企业，力争实现产值130亿元。加快建设美丽乡村。落实农村人居环境整治三年行动方案，做好新县乡村振兴示范县和其他县区乡村振兴示范片区建设。推进“厕所革命”、垃圾治理、生活污水治理和河道整治，完成100个村庄整治任务，创建特色小镇10个，建设美丽乡村示范村40个。实施农作物化肥农药使用量零增长行动，推进秸秆、畜禽粪污等资源化利用。加快“四好农村路”创建，建立以公共财政分级投入为主、社会资本和群众参与为辅的资金筹措机制，助推脱贫攻坚和乡村振兴。完成农村危房改造5560户，解决19.79万人的饮水安全问题。积极发展美丽经济，开展田园综合体建设试点，大力发展生态农业、设施农业、体验农业、定制农业，推动农业与旅游、文化、健康、养老等产业融合发展。因地制宜发展大别山区沟域经济。深化农村改革。巩固农村土地承包经营权确权登记颁证成果，完善承包地“三权分置”制度，落实好第二轮土地承包到期后再延长30年政策；稳步推进农村集体产权制度改革，全面开展农村集体资产清产核资工作，完成新县、光山县农村集体产权制度改革试点，加快建立农村资产评价体系。深入推进国家农村改革试验区“八项改革”。加强乡村治理。全面推进农业农村“领头雁”计划，引进农业技术人才，发挥乡贤作用，培养造就一支懂农业、爱农村、爱农民的“三农”工作队伍。加快乡镇文化站、农村文化广场、农家书屋等文化载体建设。依法约束贫困老人子女履行赡养义务。全面完成村“两委”换届选举工作，深入推广“四议两公开”，逐步健全自治、法治、德治相结合的乡村治理体系。积极培育勤俭节约、文明理事的乡村新风。

（六）狠抓污染防治，坚决守护蓝天碧水净土。深入贯彻习近平生态文明思想，坚持依法治污、科学治污、精准治污、全民治污，坚决打好污染防治攻坚战。

深化大气污染防治。加强国家、省市监控数据解析运用，找准污染源头，狠抓治理重点；强化过程管控，以月保季，以季保年，为采暖季留足工作空间；坚持标本兼治，加快建立中心城区大气污染网格化联防联控机制，重点抓好扬尘治理，狠抓“六控”措施落实，力争大气污染防治三项指标年度任务全面完成。加强水污染防治。明确责任分工，落实责任要求，严肃责任追究，系统推进水资源保护、水污染防治、水环境改善和水生态修复。全面落实河长制、湖长制，加强集中式饮用水水源地环境保护，加快建设市中心城区雨污分流和水污染防治工程，建成投运市第二、第三污水处理厂，全面消除市区建成区内黑臭水体，基本完成各县黑臭水体截污纳管建设和各产业集聚区污水处理厂建设，确保城市集中式饮用水水源水质达标率保持100%，境内淮河流域水质全部达到或优于Ⅲ类，重要河湖库水功能区水质达标率达到100%，全面消除劣V类水体，逐步实现“河畅、水清、湖净、岸绿、景美”的目标。开展土壤污染防治。全面落实“土十条”，开展第二次全国污染源普查和第三次全国土地调查，科学制定土壤污染管控与修复规划，全面排查危险废物产生和经营单位，动态管理土壤污染重点监管企业和疑似污染地块，加大土壤污染治理修复力度。加强生态建设。重拳出击开展河道采砂综合整治，淮河干流永久禁采、支流规范开采，坚决根治非法采砂“顽疾”。划定生态保护红线、永久基本农田、城镇开发边界控制线，加大国家级、省级自然保护区管理力度。扎实开展国家森林城市创建，完成林业生态建设98万亩。依法征收环保税，推进排污权交易市场化、公开化，进一步细化落实空气和水环境质量生态补偿机制。

（七）深化改革开放创新，充分激发发展动力活力。坚持激活内力与借助外力并重，抓住关键环节和重点领域，以改革增活力、以开放添动力、以创新促发展。

持续深化改革。推动政府机构改革，如期完成改革任务。推进国资国企改革，完成全市国有“僵尸企业”处置工作；推进市属经营性资产统一监管，加快国有企业公司制、股份制改造。加快市级政府投融资平台去行政化改革，依法依规开展市场化融资。支持金融改革创新，全面完成市县农商行组建工作。持续深化放管服改革，再承接取消下放一批行政审批事项，全面推行市场负面清单制度，严格执行新版投资项目审批流程。推进新县相对集中行政许可权改革试点。加快公共资源交易市场化改革，尽快实现全流程电子化交易。继续做好医药卫生、科技、文化、林业、盐业、粮食、供销等领域改革。扩大对外开放。加大城市整体性宣传、推介、营销力度。加强开放平台建设，建成信阳保税物流中心（B型）；加快信阳铁路口岸、淮滨水运口岸、跨境电商综合试验区等项目建设，推动开放平台功能融合；力争明港机场10月份投入运营，依托明港机场打造空港经济区；加快信阳高新区出口电子电气产品质量安全示范区建设，积极争创国家级示范区。加快培育以茶叶、电子产业和羽绒为主的出口基地，开拓“一带一路”沿线市场。深化内陆直通式海关建设和关检一体化，完善国际贸易“单一窗口”功能，提高对外开放综合服务能力。支持固始县打造面向华东的“桥头堡”。加快创新创业。抓好“四个一批”，培育发展一批创新引领型企业、人才、平台、机构。增加研究与试验发展投入，加强创新主体培育，新增高新技术企业15家，新建引进转化科技成果10项。深入推进“信阳英才计划”，实施“663青年人才工程”，加强产业工人队伍建设。支持在外能人、返乡农民工、在校大学生在信阳创新创业。加快双创载体建设，推进信阳高新区与中电光谷OVU创客星合作，打造全省一流的区域双创基地；启动海营生态智慧城规划设计；加快信阳大学科技园建设。加大力度引进一批重点新型研发机构。

（八）着力改善民生，不断满足群众美好生活需要。践行以人民为中心的发展思想，多谋民生之利，多解民生之忧，顺应人民群众新期待。

扎实办好民生实事。继续从群众最关心的现实利益问题入手，既尽力而为，又量力而行，集中精力办好以下十件实事：第一件，改扩建中心城区中小学校6所；第二件，持续实施学校（幼儿园）互联网+明厨亮灶工程；第三件，提升城乡基层医疗卫生服务质量，建设130个标准化中医馆；第四件，完成中心城区所有背街小巷整治，再打通一批断头路并对中心城区6条道路进行绿化更新；第五件，购置更新公交车200辆，新增5条公交线路；第六件，实施棚户区改造6.3万套，开工建设安置房1.67万套；第七件，新建改造46个农贸市场；第八件，建设中心城区建筑垃圾处置场；第九件，对88个深度贫困村电网进行改造升级；第十件，实现水稻生产功能区农业保险全覆盖。提高就业收入水平。深入实施全民技能振兴工程，做好高校毕业生、农村劳动力、去产能企业分流职工等重点群体就业创业工作。扎实做好退役军人安置、管理和保障工作。依法治理拖欠农民工工资问题，调整机关事业单位人员基本工资标准和社会最低工资标准。优先发展教育事业。持续推进第三期学前教育行动计划。大力推广“国家学生饮用奶计划”，扩大覆盖范围。加快“全面改薄”、城镇义务教育学校扩容和农村寄宿制学校建设，完成息县、淮滨县、商城县义务教育发展基本均衡县省级评估验收和国家认定。推进大别山高中建设，加快普及高中阶段教育。大力发展现代职业教育，深入实施职教攻坚计划二期工程。支持和规范社会力量兴办教育。加强教师队伍建设，做好教师编制统筹调整工作。不断繁荣文化事业。加大文化场馆免费开放力度，广泛开展全民阅读、百姓舞台、送戏下乡等群众性文化活动，启动信阳美术馆、信阳非物质文化遗产展览馆建设前期工作。建设文化产业示范园区和示范基地，实施文艺创作生产精品工程，培育文化市场主体，打造文化品牌。加快建设健康信阳。坚持“三医”联动，深入推进分级诊疗、家庭医生签约服务和医联体建设，推进城乡居民基本医保与大病保险制度有效衔接，全面推行按病种付费为主的多元复合式医保支付方式，积极探索公立医院薪酬制度和员额制改革。深入开展爱国卫生运动。持续提升医疗服务和安全管理水平。广泛开展全民健身活动，继续办好群众性体育赛事。实施食品安全战略，积极争创国家级食品安全示范市。健全社会保障体系。全面实施全民参保计划，努力实现户籍人口应保尽保。适度提高城乡低保和优抚补助标准，积极落实养老保险全国统筹。加快救助申请家庭经济状况核对信息平台建设，实现社会救助“一门受理、协同办理”。推进残疾人精准康复服务行动，健全农村留守儿童和妇女、老年人关爱服务体系。保障群众住有所居。加快建立多主体供给、多渠道保障、租购并举的住房制度，满足群众多层次居住需求。培育发展住房租赁市场，扩大公租房保障范围。推进住房公积金归集扩面，完善使用政策，扩大受益范围。推进社会治理创新。完善安全生产责任制，健全公共安全体系，强化重点行业领域安全专项治理，确保实现“一个杜绝、三个持续下降”目标。完善矛盾纠纷预防化解体系和立体化社会治安防控体系，深入开展扫黑除恶专项斗争。防范化解风险隐患。开展互联网金融和非法集资风险排查，加大金融知识宣传普及力度，完善金融风险防控体系，确保不发生系统性风险。稳妥化解企业风险，深入推进市场化“债转股”。持续开展政府性债务清理，加强政府债务风险评估和监测预警，打好防范化解重大风险攻坚战。

加强国防教育、国防动员和国防后备力量建设，支持军队改革，支持驻信解放军、武警部队、民兵和人民防空建设，深入开展双拥共建，巩固军政军民团结。支持工会、共青团、妇联和科协、工商联、社科联、红十字会等人民团体更好发挥桥梁纽带作用，全面落实民族宗教政策，做好外事侨务、对台工作。继续抓好统计、物价、邮政、通信、地质、气象、档案、地方志、防震减灾、机关事务、市场发展、无线电管理等工作。开展好第四次全国经济普查。

（九）抓好要素运作，保障经济社会持续健康发展。紧扣关键要素供给，创新思路，改进方法，完善机制，打牢高质量发展保障基础。

抓好财源建设。深化财税金融改革，细分奖励政策，大力支持招商引资和后续财源培植。优化财政资金配置，严格执行零基预算，强化财政专项资金和政府投资项目监管，集中财力保基本、保运转、保民生、保重点。持续抓好“三块地”改革，完成浉河三期等重点区域的土地收储；加大农村空闲、废弃的宅基地拆旧复垦力度，用足用活城乡增减挂钩政策；推进土地综合整治，高标准规划设计补充耕地项目。提升融资能力，积极向上争取财政支持；规范开展PPP工作，支持在水务、环境保护、交通运输、市政工程等行业规范开展PPP项目资产证券化。提高落地实效。充分利用“互联网+政务服务”平台，整合行政审批权，精简审批要件，优化审批流程，纵深推进“多证合一”改革，力争做到“只进一扇门”“最多跑一次”。采取以规划代立项、多规合一、联合图审、统一验收、联审联批等措施，提高审批效率。完善领导分包、现场会商等重大项目推进机制，促进项目投产达产。做好跟踪服务。深入开展“帮促”活动，落实重点企业“首席服务员”制度，及时协调解决企业问题。分片区统筹研究解决企业历史遗留问题。设立多类别的产业基金、创新创业基金和引导基金，开展定向、定岗和订单式职业技能培训，帮助企业解决融资和用工难题。大力弘扬企业家精神，尊重、关心、保护企业家，注重企业人才培养和企业家队伍建设，建立重点企业“直通车”制度，让企业家放心干、放手干，营造激励企业家干事创业的良好氛围。

三、2018年政府自身建设

扎实开展“责任落实年”活动，造就信念过硬、政治过硬、责任过硬、能力过硬、作风过硬的干部队伍，努力建设人民满意的政府。

（一）提升政治站位。牢固树立“四个意识”，增强“四个自信”，坚决维护习近平总书记核心地位，坚决维护党中央权威和集中统一领导，在政治立场、政治方向、政治原则、政治道路上同以习近平同志为核心的党中央保持高度一致，决不搞上有政策、下有对策，自觉做到令行禁止。持续推进“两学一做”学习教育常态化制度化，认真开展“不忘初心、牢记使命”主题教育。

（二）强化责任担当。大力弘扬“焦裕禄精神”，保持对群众的亲劲、抓工作的韧劲、干事业的拼劲，做到守土有责、守土负责、守土尽责。深入推进政务督查，强化效能问责和行政问责，大力整治“门好进、脸好看、事难办”“为了不出事，宁可不干事”等不作为、慢作为问题。建立健全正向激励机制和容错纠错机制，严格落实“三个区分开来”，进一步激励广大干部新时代新担当新作为。

（三）增强工作本领。着力提升“八种本领”，大兴学习之风、调研之风、干事之风。注重更新知识层次和思维方式，运用专业化团队推进工作。学会站在企业和老百姓的角度考虑问题，主动作为，贴心服务，不为无为找借口，只为能为找路子。推行“一线工作法”，不断提升政府执行力。

（四）坚持依法行政。恪守宪法法律，把法治思维和法治方式贯穿政府工作全过程，切实按法定权限用权，不逾越法律界线。深入推进政务公开，让权力在阳光下运行。自觉接受人大、政协和社会监督，认真办理人大代表建议和政协委员提案。完善政府法律顾问制度，落实行政执法责任制。

（五）保持清正廉洁。全面落实从严治党主体责任，坚决履行党风廉政建设“一岗双责”，坚持党风廉政建设和政府工作同部署、同安排、同考核。深入贯彻党内政治生活准则和党内监督条例，严格落实中央八项规定精神和实施细则，驰而不息整治“四风”。加强审计监督，从源头上预防腐败。坚守节用裕民的正道，“三公”经费只减不增，把有限的资源和财力用在推动发展、改善民生上。

各位代表！梦想照亮前方，奋斗赢得未来。时代的问卷交到了我们手上，我们作为新时代的答卷人，唯有苦干实干，才能不负重托。让我们高举习近平新时代中国特色社会主义思想的伟大旗帜，在省委、省政府和市委的坚强领导下，务实作为，锐意进取，向全市人民交上一份满意的答卷!
